# Multidimensional Visualization and AI-Driven Prediction Using Clinical and Biochemical Biomarkers in Premature Cardiovascular Aging

**DOI:** 10.3390/biomedicines13102482

**Published:** 2025-10-12

**Authors:** Kuat Abzaliyev, Madina Suleimenova, Symbat Abzaliyeva, Madina Mansurova, Adai Shomanov, Akbota Bugibayeva, Arai Tolemisova, Almagul Kurmanova, Nargiz Nassyrova

**Affiliations:** 1 Department of Internal Medicine, Faculty of Medicine and Healthcare, Al-Farabi Kazakh National University, Almaty 050040, Kazakhstan; 2 Department of Big Data and Artificial Intelligence, Faculty of Information Technology, Al-Farabi Kazakh National University, Almaty 050040, Kazakhstan; 3 Department of Postdoctoral Research, Nazarbayev University, Astana 010000, Kazakhstan; 4 LLP «International Research Institute for Postgraduate Education», Almaty 050060, Kazakhstan; 5 Department of Science and International Relations, Non-State Educational Institution «Kazakh-Russian Medical University», Almaty 050040, Kazakhstan

**Keywords:** biomarkers, premature aging, parallel coordinates, t-SNE, principal component analysis, machine learning, random forest

## Abstract

**Background:** Cardiovascular diseases (CVDs) remain the primary cause of global mortality, with arterial hypertension, ischemic heart disease (IHD), and cerebrovascular accident (CVA) forming a progressive continuum from early risk factors to severe outcomes. While numerous studies focus on isolated biomarkers, few integrate multidimensional visualization with artificial intelligence to reveal hidden, clinically relevant patterns. **Methods:** We conducted a comprehensive analysis of 106 patients using an integrated framework that combined clinical, biochemical, and lifestyle data. Parameters included renal function (glomerular filtration rate, cystatin C), inflammatory markers, lipid profile, enzymatic activity, and behavioral factors. After normalization and imputation, we applied correlation analysis, parallel coordinates visualization, *t*-distributed stochastic neighbor embedding (t-SNE) with k-means clustering, principal component analysis (PCA), and Random Forest modeling with SHAP (SHapley Additive exPlanations) interpretation. Bootstrap resampling was used to estimate 95% confidence intervals for mean absolute SHAP values, assessing feature stability. **Results:** Consistent patterns across outcomes revealed impaired renal function, reduced physical activity, and high hypertension prevalence in IHD and CVA. t-SNE clustering achieved complete separation of a high-risk group (100% CVD-positive) from a predominantly low-risk group (7.8% CVD rate), demonstrating unsupervised validation of biomarker discriminative power. PCA confirmed multidimensional structure, while Random Forest identified renal function, hypertension status, and physical activity as dominant predictors, achieving robust performance (Accuracy 0.818; AUC-ROC 0.854). SHAP analysis identified arterial hypertension, BMI, and physical inactivity as dominant predictors, complemented by renal biomarkers (GFR, cystatin) and NT-proBNP. **Conclusions:** This study pioneers the integration of multidimensional visualization and AI-driven analysis for CVD risk profiling, enabling interpretable, data-driven identification of high- and low-risk clusters. Despite the limited single-center cohort (*n* = 106) and cross-sectional design, the findings highlight the potential of interpretable models for precision prevention and transparent decision support in cardiovascular aging research.

## 1. Introduction

Cardiovascular diseases (CVDs) remain the primary cause of death globally, accounting for approximately 17.9 million deaths annually, according to the World Health Organization [1]. Among the numerous clinical entities comprising CVDs, arterial hypertension, ischemic heart disease (IHD), and cerebrovascular accident (CVA) are of paramount clinical and public health importance. Hypertension is recognized as one of the most prevalent and modifiable cardiovascular risk factors, affecting over 1.28 billion adults worldwide. Persistently elevated blood pressure contributes to structural and functional vascular changes that predispose individuals to coronary artery disease and stroke.

IHD, particularly in patients requiring stent placement, represents an advanced stage of coronary atherosclerosis with significant myocardial ischemia. It is a major cause of morbidity, often associated with recurrent hospitalizations and long-term pharmacological management. CVA, in turn, is one of the most severe neurological outcomes of cardiovascular pathology, resulting from acute disruption of cerebral blood flow. Both IHD and CVA share common risk factors, including hypertension, dyslipidemia, impaired renal function, and lifestyle-related determinants such as smoking, physical inactivity, and unhealthy diet.

While numerous studies have addressed individual risk factors or specific outcomes, fewer have simultaneously examined the continuum from early-stage risk factor manifestation (hypertension) to late-stage cardiovascular events (IHD and CVA) within the same patient population. Such an approach enables a comprehensive understanding of shared biomarker and lifestyle patterns as well as outcome-specific features, thereby providing insights into the progression and interrelation of cardiovascular pathologies.

Studies show that the most significant risk factors for CVD are age, gender, chronic diseases such as diabetes mellitus (DM) and chronic kidney disease (CKD), as well as behavioral factors such as smoking and alcohol consumption [2]. At the same time, various clinical and laboratory parameters, including body mass index (BMI), low-density lipoprotein (LDL) levels, triglycerides (TG), and glomerular filtration rate (GFR), can serve as indicators of increased risk [3]. With the development of artificial intelligence technologies, including deep learning, new opportunities have emerged for more accurate disease prediction based on a variety of clinical and biochemical data. The use of artificial neural networks for processing large amounts of data significantly improves the accuracy of predictions and the identification of hidden dependencies between various risk factors [4]. Neural networks have already proven their effectiveness in predicting cardiovascular diseases, where traditional methods of statistical analysis do not always provide the necessary accuracy [5]. Study [6] presents a systematic review of the application of artificial intelligence (AI) methods for predicting cardiovascular disease (CVD). Sixty studies using machine learning (ML) and deep learning (DL) on clinical, genetic, and wearable device data were analyzed. Among the most effective algorithms are random forest (RF), support vector machines (SVM) and convolutional neural networks (CNN), with an accuracy of up to 100%. The authors note the lack of integration of heterogeneous data, the complexity of model interpretation and the limited sample size, which reduces applicability in clinical practice. Areas for improvement have been proposed, including model integration and the use of multi-modal data. Study [7] analyses contemporary technological advances in the diagnosis of cardiovascular diseases (CVD) with a focus on the integration of artificial intelligence (AI), machine learning (ML), image analysis, and telemedicine. A bibliometric analysis of publications from the SCOPUS database using PRISMA-ScR revealed a growing interest in digital technologies in cardiology since 2018. Key trends include the application of AI in precision medicine, the use of 3D printing, and multi-omeric analysis. The authors note the potential of AI to improve diagnosis and prognosis but emphasize the need for clinical validation and resolution of data interoperability issues. A recent study of Shah [8] introduced a hybrid ensemble learning framework combining Gradient Boosting, CatBoost, and Neural Networks in a stacked architecture to predict cardiovascular disease risk. Using public datasets, the model integrated clinical and lifestyle factors (blood pressure, BMI, cholesterol–glucose ratio, etc.) and applied explainable AI tools (SHAP, t-SNE, PCA) for feature interpretation. The approach achieved AUC-ROC = 0.82, with balanced performance (Precision 81%, Recall 83%, F1-score 82%). The authors emphasize that combining strong predictive accuracy with interpretability can enhance clinical trust and support targeted prevention strategies. Moreover, in the next investigation [9] a scoping review of 17 studies examined visualization techniques for estimating cardiovascular disease (CVD) risk. Commonly used risk factors included age, sex, blood pressure, smoking, diabetes, and cholesterol profiles. The most frequent visualization methods were visual cues, bar charts, and graphs. While digital health tools can enhance understanding, motivation, and compliance by integrating machine learning and visual analytics, visualization is rarely embedded directly into prognostic models. Evidence on mobile health’s direct impact on CVD outcomes remains limited. By Salah [10] a study using data from 14,083 participants in the Add Health cohort developed the first explainable ML framework for predicting long-term CVD risk from adolescence. Four models (DT, RF, XGBoost, DNN) were tested, with XGBoost performing best (AUC-ROC = 84.5%, AUC-PR = 96.9%). Using explainable AI, key adolescent predictors were identified, showing that early-life data can effectively forecast adult CVD risk, supporting primordial prevention and personalized interventions. A study from the CLAVIS-COVID registry [11] examined 601 COVID-19 patients with cardiovascular disease and/or risk factors (CVDRF) to assess the prognostic value of the neutrophil-to-lymphocyte ratio (NLR). Higher baseline NLR quartiles were associated with increased in-hospital mortality; however, baseline NLR was not an independent prognostic factor in multivariate analysis (AUC = 0.682). Follow-up NLR showed stronger predictive power (AUC = 0.893) and, when visualized via t-SNE, highlighted a distinct high-mortality cluster. These findings suggest that the prognostic utility of NLR in this population depends on the timing of measurement. Moreover, according to Lu W. [12] the Digital Brain (DB) is a large-scale spiking neural network simulation platform based on personalized MRI data and biological constraints. It models up to 86 billion neurons and 47.8 trillion synapses using 14,012 GPUs with a two-level routing scheme to speed up spike transmission. The DB accurately reproduces resting-state brain activity (BOLD signals) and responds to perceptual inputs in tasks, demonstrating the feasibility of creating a functional digital replica of the human brain for diverse applications. Recent work by Kolk H. [13] demonstrated that integrating late gadolinium-enhanced cardiac MRI, 12-lead ECG, and clinical data in a multimodal deep learning model significantly improved prediction of malignant ventricular arrhythmias in patients with non-ischaemic systolic heart failure (AUROC = 0.84), outperforming single-modality approaches. This highlights the prognostic advantage of combining heterogeneous clinical and imaging modalities for arrhythmic risk stratification. The study [14] proposes a novel approach to predicting cardiovascular disease (CVD) risk in adults aged 65+ by integrating clinical, biochemical, and immunological markers with mathematical modeling and machine learning. Data included immune cell indices, cytokines, cardiovascular and inflammatory markers, metabolic indicators, organ function parameters, and lifestyle factors. Numerical modeling methods (Runge–Kutta, Adams–Bashforth, backward Euler) were applied to track biomarker dynamics. Key predictors of aging were HLA-DR (50%), CD14 (41%), and CD16 (38%); BMI correlated with placental growth factor, while physical activity was positively linked to glomerular filtration rate and inversely to SOD activity. The model enhances early CVD risk detection and supports personalized prevention strategies.

Therefore, the aim of this study is to develop and validate an interpretable artificial intelligence framework that integrates advanced multidimensional visualization techniques—Parallel Coordinates, t-distributed Stochastic Neighbor Embedding (t-SNE), and Principal Component Analysis (PCA)—with Random Forest–based predictive modeling to identify shared and disease-specific biomarker patterns in cardiovascular diseases. This approach seeks to combine high predictive performance with transparent clinical interpretability, enabling early risk stratification, precision prevention strategies, and data-driven decision support in cardiology.

## 2. Materials and Methods

The study was conducted within the framework of the scientific and technical project №AP19677754 ‘Development of markers and diagnostic algorithm for detection and prevention of early cardiovascular aging’, State Institution “Ministry of Science and Higher Education of the RK”, Almaty, Kazakhstan.

A cohort of 106 individuals aged ≥65 years was analyzed as part of a study on premature cardiovascular aging.

Exclusion criteria included HIV infection, a history of tuberculosis, any acute infectious disease within three months prior to enrollment, mental disorders limiting the ability to provide adequate cooperation, any diagnosed allergic reaction, and refusal to participate in the study. All participants received comprehensive information regarding the study’s objectives, methodology, and conditions, after which they voluntarily provided written informed consent to participate.

For biochemical analysis, 3–5 mL of venous blood was collected from each patient into vacuum tubes without anticoagulant and delivered to the clinical diagnostic laboratory within 2 h. Biochemical parameters were measured using an automated biochemical analyzer (Roche Diagnostics, Basel, Switzerland) following the manufacturer’s protocols. Commercial reagent kits were employed to determine the levels of albumin, C-reactive protein (CRP), pronatriuretic peptide (NT-pro-BNP), serum cholinesterase, cystatin C, and superoxide dismutase. All results were documented in the electronic laboratory information system and subsequently used for statistical analysis.

The dataset included the following indicators as biomarkers of cardiovascular aging and as measures of the respondents’ social status (see Table 1 and Table 2):

The integrated dataset included clinical, biochemical, and behavioral indicators. Missing continuous values were imputed using median replacement, while categorical variables were binary encoded. For visualization purposes (parallel coordinates, PCA, and t-SNE), Min–Max normalization was applied to scale features between 0 and 1. For machine learning analysis, unscaled data were used since tree-based models such as Random Forest are invariant to feature scaling.

Data analysis followed a multimethod pipeline that combined statistical, visualization, and AI-based approaches. Pearson correlation coefficients were computed to identify associations among clinical and biochemical variables. Parallel coordinates, principal component analysis (PCA), and t-distributed stochastic neighbor embedding (t-SNE) were employed to explore multidimensional relationships between biomarkers and lifestyle factors. To identify potential subgroups, k-means clustering (k = 2) was performed on the t-SNE embedding, and the silhouette score (0.72) confirmed satisfactory separation between clusters.

Feature relevance was evaluated using a Random Forest classifier consisting of 500 trees with balanced class weights to account for class imbalance. The dataset was randomly divided into training (75%) and testing (25%) subsets with stratified sampling to preserve outcome proportions. Model performance was assessed using accuracy, precision, recall, F1-score, and area under the receiver operating characteristic curve (AUC-ROC). To enhance transparency and interpretability, SHapley Additive exPlanations (SHAP) were applied to decompose model predictions into additive feature contributions. Global feature importance was quantified by mean absolute SHAP values, while individual contributions were visualized using beeswarm and bar plots. To ensure robustness and account for sample uncertainty, bootstrap resampling (*n* = 1000) was used to estimate 95% confidence intervals for mean absolute SHAP values, thereby quantifying the stability of each predictor’s contribution.

All computations were performed in Python 3.10 using pandas, numpy, scikit-learn, matplotlib, and seaborn.

## 3. Results and Discussion

**Figure 1:**
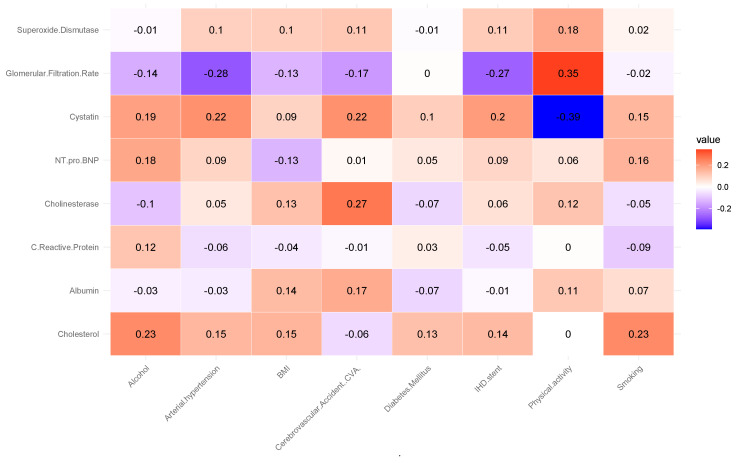
Correlation coefficient of the biochemical biomarkers of all experimented patients.

Correlation analysis revealed several notable associations (see Figure 1).

The analysis revealed a moderate positive correlation between glomerular filtration rate (GFR) and physical activity (r = 0.35). This suggests that individuals with higher levels of physical activity tend to have better renal function, as reflected by higher GFR values. This association aligns with previous findings indicating that regular physical activity can improve cardiovascular and renal health by reducing oxidative stress, improving endothelial function, and lowering blood pressure. A positive correlation was observed between cholinesterase levels and the occurrence of cerebrovascular accidents (CVA) (r = 0.27). It may indicate that alterations in cholinesterase activity are linked to vascular pathophysiology affecting cerebral circulation. Elevated or dysregulated cholinesterase levels have been reported in some studies as potential markers of systemic inflammation and metabolic disturbances, which may contribute to an increased risk of ischemic events. Cholesterol levels showed a positive correlation with alcohol consumption and smoking status (r = 0.23). This association, although modest, is consistent with epidemiological evidence indicating that lifestyle factors such as alcohol intake and tobacco use can adversely affect lipid metabolism, leading to elevated total cholesterol levels. These behaviors are well-established risk factors for atherosclerosis and cardiovascular disease, suggesting that their combined impact may exacerbate dyslipidemia and related health outcomes.

A moderate negative correlation was found between cystatin C levels and physical activity (r = −0.39). Higher levels of physical activity were associated with lower concentrations of cystatin C, a biomarker of renal dysfunction and cardiovascular risk. This inverse relationship supports the notion that regular physical activity may help preserve kidney function and reduce systemic inflammation, thereby lowering cystatin C levels. Such findings agree with studies highlighting the protective effects of exercise on renal and cardiovascular health. An inverse correlation was observed between glomerular filtration rate (GFR) and arterial hypertension (r = −0.28). This suggests that individuals with elevated blood pressure tend to have reduced renal filtration capacity, reflecting early manifestations of hypertensive nephropathy. Chronic hypertension is known to cause structural and functional changes in the renal vasculature, leading to progressive decline in GFR. This finding reinforces the established link between hypertension and impaired kidney function, highlighting the importance of blood pressure control in preventing renal deterioration. A negative correlation was identified between glomerular filtration rate (GFR) and ischemic heart disease (IHD) (r = −0.27). Lower GFR values were associated with a higher prevalence of IHD, suggesting that impaired renal function may be linked to increased cardiovascular risk. This relationship is consistent with the cardiorenal syndrome concept, where deterioration in kidney function contributes to the progression of atherosclerosis, endothelial dysfunction, and myocardial ischemia. These findings emphasize the interconnected nature of renal and cardiovascular health.

Parallel coordinates visualization was employed to assess the multidimensional patterns of biochemical, clinical, and lifestyle variables in relation to three major cardiovascular outcomes: arterial hypertension, cerebrovascular accident (CVA), and ischemic heart disease with stent placement (IHD/stent).

In all three analyses, consistent trends emerged. Patients in the outcome-positive groups displayed markedly reduced glomerular filtration rate (GFR) and elevated cystatin C levels, reflecting impaired renal function. Physical activity was frequently lower among these patients compared with controls. Arterial hypertension was almost universal in the IHD/stent group and highly prevalent among CVA patients, further supporting its role as a major cardiovascular risk factor.

**Figure 2:**
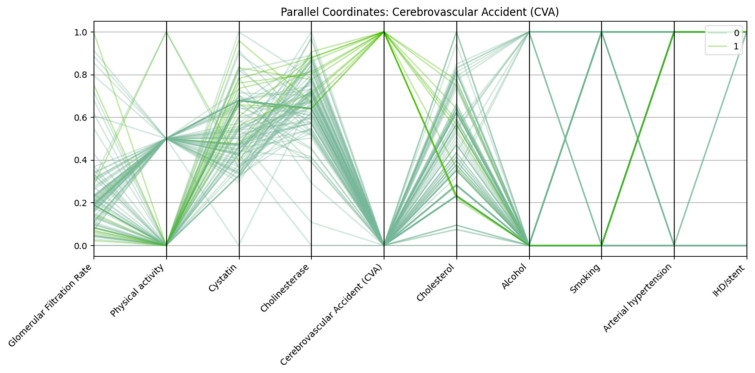
Parallel coordinates plot of selected biomarkers and risk factors for patients with and without cerebrovascular accident (CVA).

Each vertical axis in Figure 2 represents a normalized variable, including renal function markers (glomerular filtration rate, cystatin C), biochemical parameters (cholinesterase, cholesterol), and lifestyle/clinical factors (physical activity, alcohol use, smoking, arterial hypertension, ischemic heart disease/stent). Patients with CVA (class “1”) generally exhibit lower GFR, higher cystatin C levels, reduced physical activity, and a higher prevalence of arterial hypertension compared with those without CVA (class “0”).

There is also a link between stroke and alcohol consumption, which may indicate additional behavioral risk factors.

**Figure 3:**
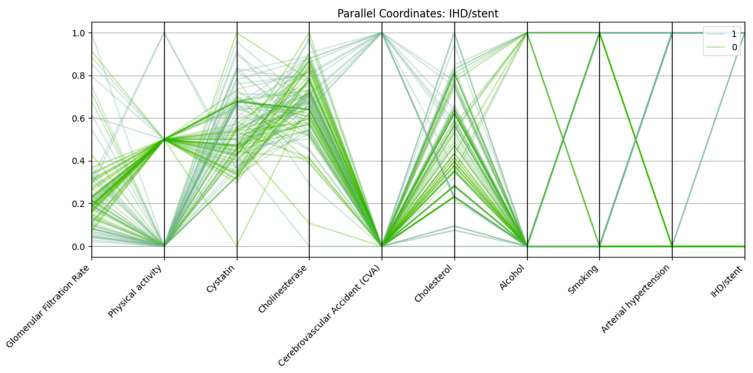
Parallel coordinates plot of selected biomarkers and risk factors for patients with and without ischemic heart disease/stent (IHD/stent).

Patients with IHD/stent (class “1”) in Figure 3 show lower GFR, elevated cystatin C levels, nearly universal arterial hypertension, and a higher prevalence of CVA compared with those without IHD/stent (class “0”). Physical activity is reduced in many cases, while cholesterol and cholinesterase levels are often slightly higher in the IHD/stent group.

Patients with IHD and stenting have:

- Lower glomerular filtration rate;
- Elevated cystatin and cholesterol levels;
- High frequency of concomitant hypertension;
- Low level of physical activity;
- More frequent alcohol consumption and a history of smoking.

The data confirm the cumulative nature of risk factors in the development of severe forms of cardiovascular disease.

**Figure 4:**
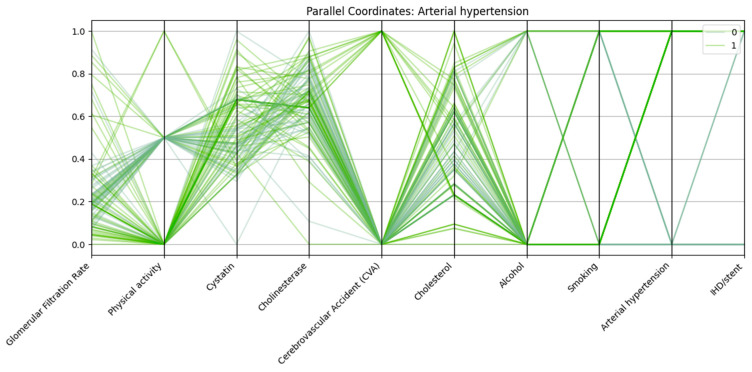
Parallel coordinates plot of selected biomarkers and risk factors for patients with and without arterial hypertension.

Each vertical axis in Figure 4 represents a normalized variable, including renal markers (glomerular filtration rate, cystatin C), biochemical parameters (cholinesterase, cholesterol), and lifestyle/clinical factors (physical activity, alcohol use, smoking, cerebrovascular accident, ischemic heart disease/stent). Patients with arterial hypertension (class “1”) generally exhibit lower GFR, higher cystatin C levels, reduced physical activity, and a higher prevalence of ischemic heart disease/stent and cerebrovascular accident compared with those without hypertension (class “0”). In addition, a significant proportion of patients with hypertension demonstrate low physical activity and are more likely to have a history of concomitant cardiovascular events.

t-SNE visualizations showed partial clustering of patients according to outcome, indicating that biomarker-lifestyle patterns carry discriminative information, but with overlap reflecting shared risk factors. PCA projections captured 42–47% of total variance in the first two components, revealing both common and outcome-specific variance structures.

**Figure 5:**
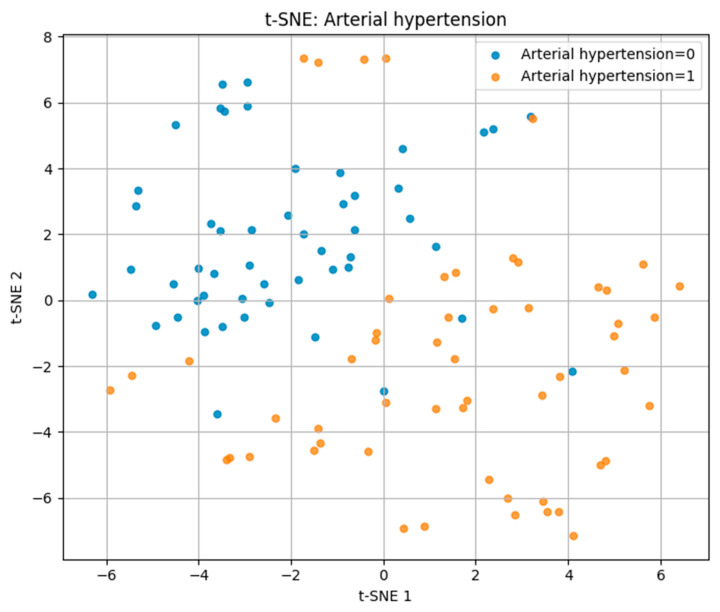
t-SNE plot for arterial hypertension.

For arterial hypertension, the t-SNE plot in Figure 5 revealed a partial separation between hypertensive (label = 1) and normotensive (label = 0) patients. While some degree of overlap was present, hypertensive cases tended to cluster on the right side of the projection space, suggesting distinguishable multidimensional profiles driven by key risk factors.

**Figure 6:**
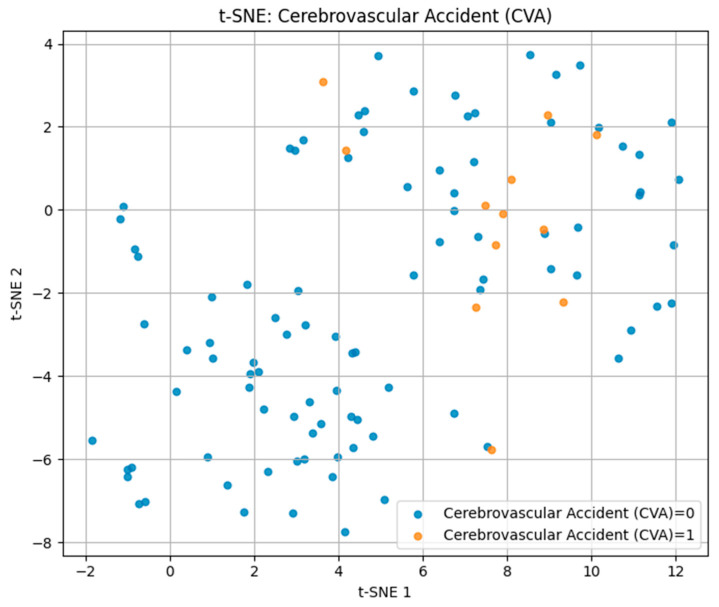
t-SNE plot for cerebrovascular accident (CVA).

For cerebrovascular accident (CVA) in Figure 6, positive cases were sparsely distributed among the negative group without clear cluster formation, indicating a high degree of heterogeneity in biomarker and lifestyle patterns. This dispersion aligns with the multifactorial and variable nature of stroke pathophysiology.

**Figure 7:**
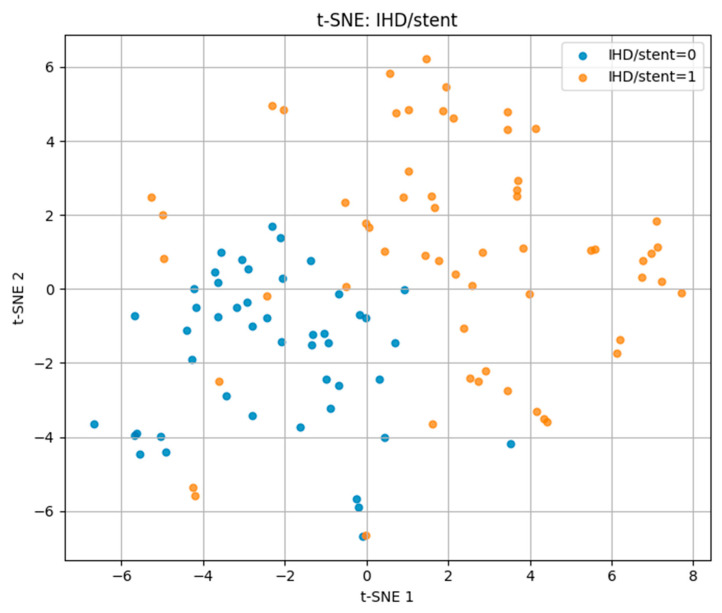
t-SNE plot for IHD/stent.

For ischemic heart disease with stent placement (IHD/stent) in Figure 7, the t-SNE projection showed more distinct clustering compared to the other outcomes. Positive cases occupied a largely separate region of the projection space, with minimal overlap with negative cases. This suggests that patients with IHD/stent share more homogeneous risk profiles, making them more separable in the multidimensional feature space.

These findings demonstrate that t-SNE can visually capture differences in multidimensional risk factor patterns, with separability being highest for IHD/stent, moderate for hypertension, and lowest for CVA.

**Figure 8:**
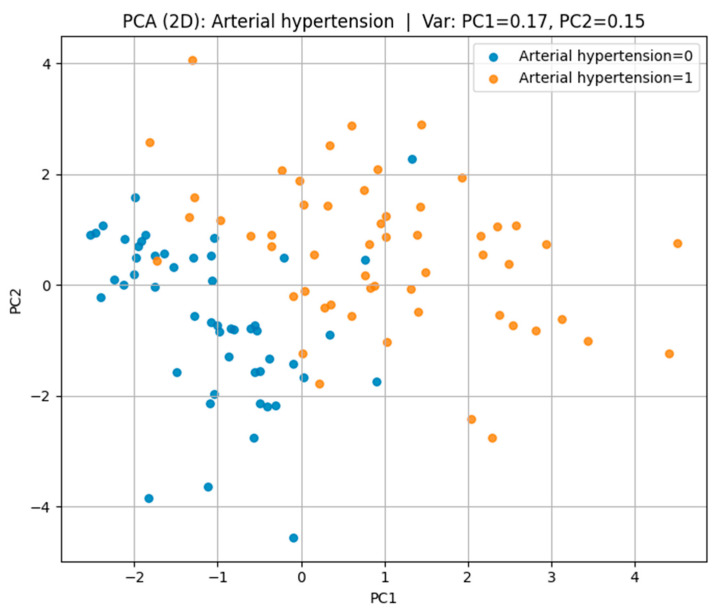
PCA plot for arterial hypertension.

Principal Component Analysis (PCA) in Figure 8 was employed to reduce the dimensionality of the dataset and to visualize its underlying structure while retaining the maximum possible variance.

In the case of arterial hypertension, the first two principal components accounted for 32% of the total variance (PC1 = 17%, PC2 = 15%), revealing a partial separation between hypertensive and non-hypertensive groups in the feature space.

**Figure 9:**
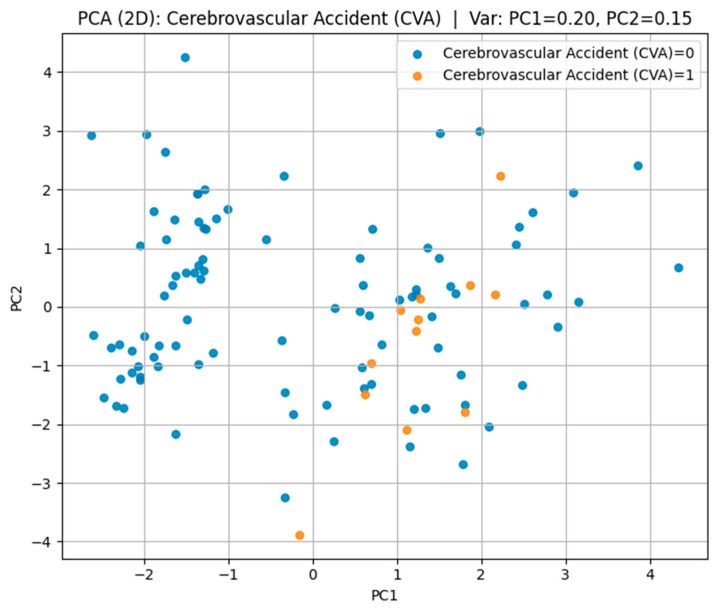
PCA plot for cerebrovascular accident.

For cerebrovascular accident (CVA) in Figure 9, the first two components explained 35% of the variance (PC1 = 20%, PC2 = 15%); however, substantial overlap between the groups was observed, suggesting a high degree of heterogeneity in their multidimensional profiles.

**Figure 10:**
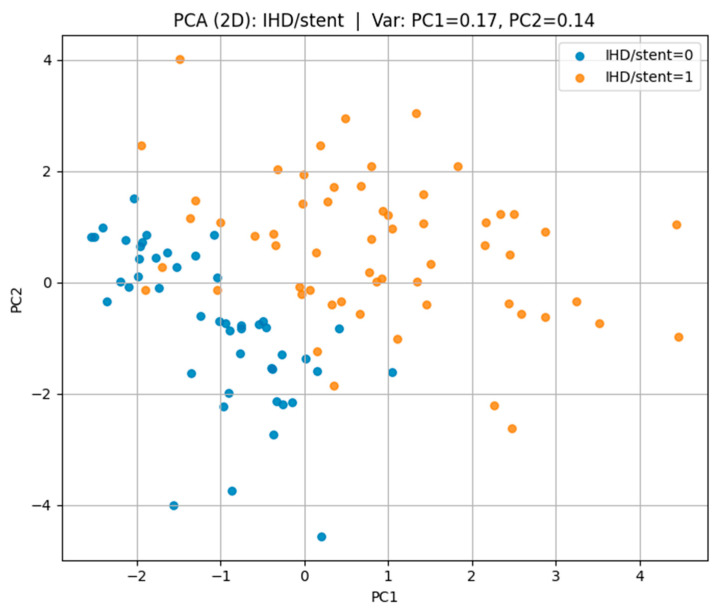
PCA plot for IHD/stent.

Regarding ischemic heart disease with stent placement (IHD/stent) in Figure 10, the first two principal components captured 31% of the variance (PC1 = 17%, PC2 = 14%), with a more distinct separation between the affected and non-affected groups, indicating more homogeneous risk factor patterns among cases.

**Figure 11:**
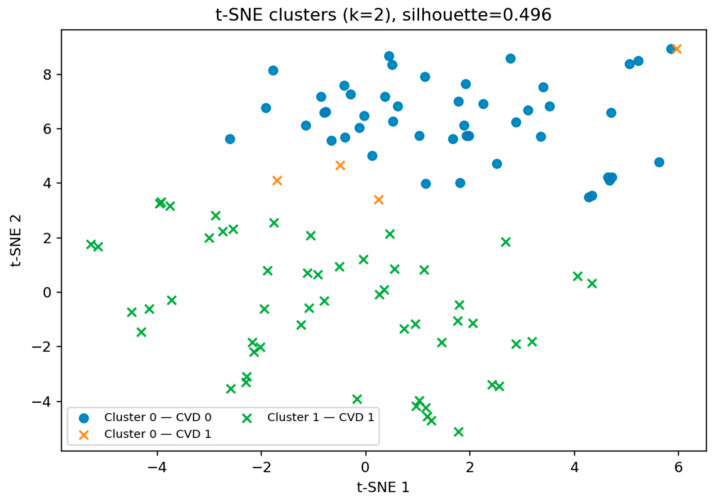
t-SNE projection of participants.

After performing the initial t-SNE visualization, we applied principal component analysis (PCA) to capture the maximal variance in the dataset using a reduced number of dimensions. While PCA revealed partial separation between groups, its linear nature may limit the ability to capture non-linear patterns. Therefore, to further investigate potential hidden subgroup structures, we returned to the t-SNE representation and applied k-means clustering in the reduced space (see Figure 11). This allowed us to identify two distinct biomarker patterns, with a moderate silhouette score of 0.496, partially corresponding to the presence or absence of cardiovascular disease (CVD).

The t-SNE clustering results in Table 3 revealed two markedly distinct participant groups. One cluster (Cluster 1) contained only CVD-positive individuals, achieving a 100% disease rate, while the other (Cluster 0) represented a predominantly low-risk profile, with only 7.8% of participants having CVD. This complete separation of high- and low-risk groups suggests that the combination of biochemical and clinical biomarkers captures disease-specific patterns with high discriminatory power.

From a clinical perspective, such separation has important implications for early risk stratification. Individuals with a biomarker profile like Cluster 1 may require more aggressive monitoring, lifestyle intervention, or preventive therapy, while those in Cluster 0 could potentially follow less intensive follow-up schemes. Importantly, the small number of CVD-positive cases within the low-risk cluster suggests the possibility of subtypes of CVD with distinct pathophysiological mechanisms, warranting further investigation.

**Figure 12:**
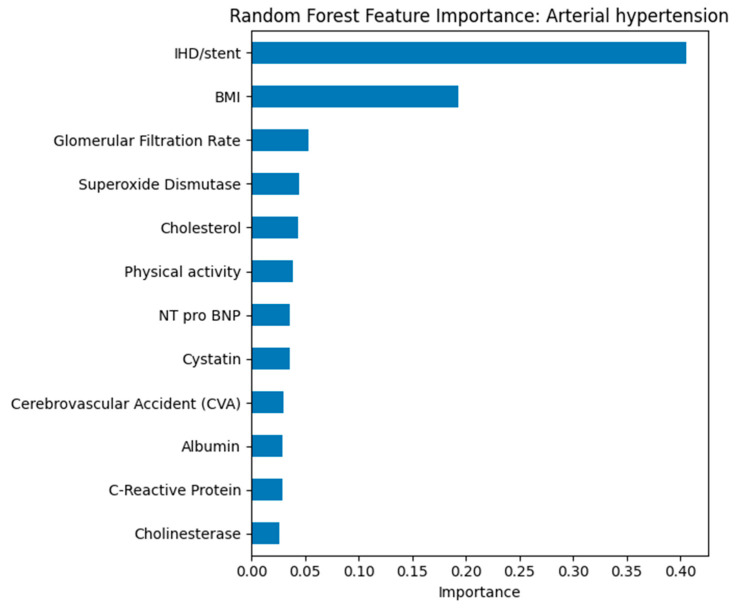
Random Forest feature importance for predicting arterial hypertension.

**Figure 13:**
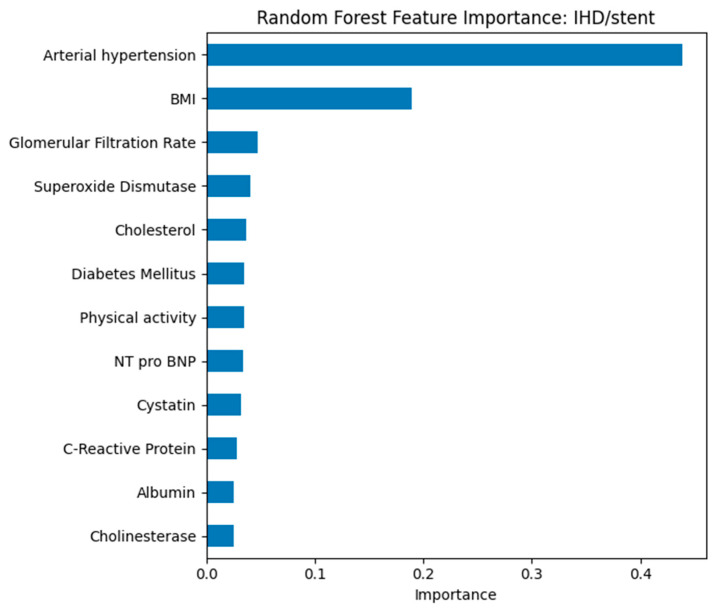
Random Forest feature importance for predicting ischemic heart disease (IHD)/stent presence.

**Figure 14:**
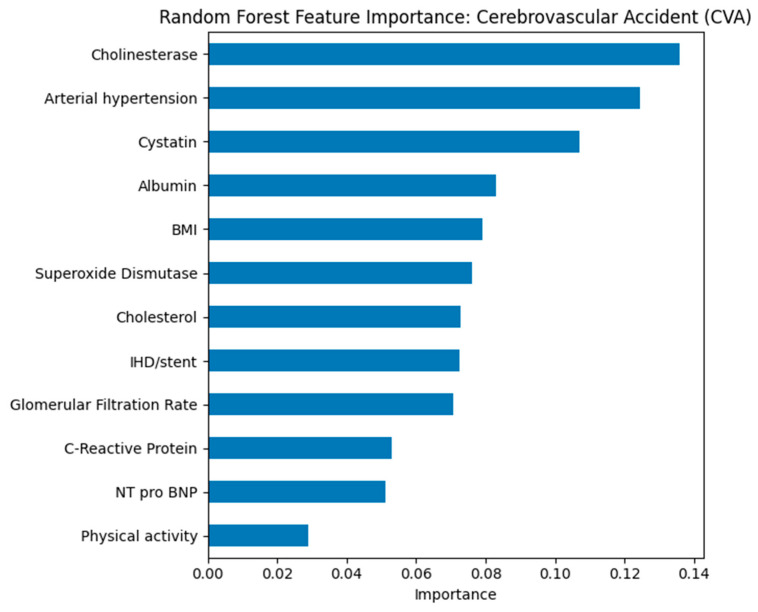
Random Forest feature importance for predicting cerebrovascular accident (CVA).

Random Forest feature importance ranked GFR, cystatin C, hypertension status, and physical activity as top predictors across all outcomes (see Figure 12). For IHD/stent (see Figure 13), cholesterol and CRP also ranked highly, while for CVA (see Figure 14), cholinesterase and albumin contributed more prominently.

The presence of IHD/stenting and body mass index were the most significant predictors of arterial hypertension. The high contribution of IHD/stenting reflects the close clinical relationship between hypertension and coronary artery disease. Body mass index confirms the key role of obesity in the pathogenesis of hypertension. Other biomarkers, including glomerular filtration rate, cholesterol, and cystatin, have less weight but indicate the multifactorial nature of the disease.

In the model for predicting stroke, cholinesterase, arterial hypertension, and cystatin showed the greatest significance. The high role of cholinesterase may reflect its connection with inflammatory and metabolic processes that affect cerebrovascular risk. Arterial hypertension is confirmed as one of the key factors in the development of stroke. Cystatin and indicators of kidney function, including glomerular filtration rate, point to the importance of chronic kidney dysfunction in the pathogenesis of the disease. Thus, the combination of hypertension, impaired kidney function, and altered protein metabolism may serve as a predictor of high CVA risk.

In the model for predicting ischemic heart disease with stenting, arterial hypertension and body mass index were found to be the most significant factors. Arterial hypertension is confirmed as a key factor contributing to the development of coronary pathology and the need for interventional treatment. Body mass index again emerges as the leading modifiable risk factor. Additional markers, including glomerular filtration rate, cholesterol level, presence of diabetes mellitus, and physical activity indicators, contribute less but reflect the multifactorial nature of the disease.

**Figure 15:**
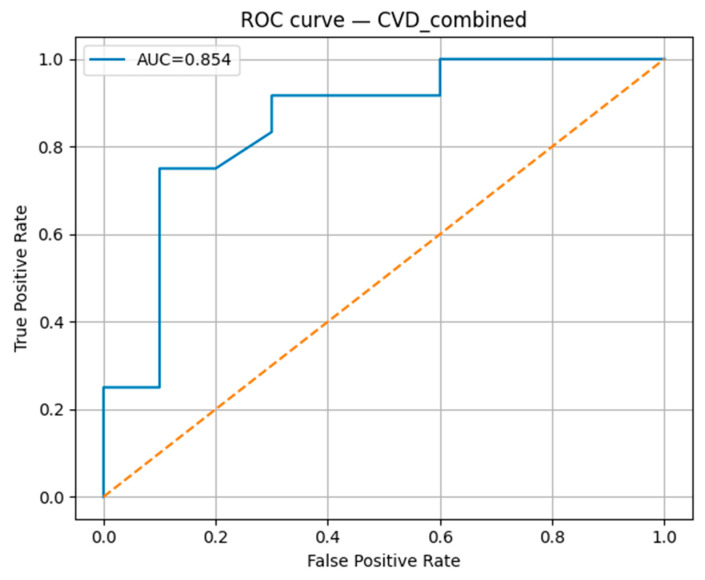
ROC curve for the combined CVD prediction model.

The Random Forest classification model demonstrated robust predictive performance for the studied cardiovascular outcomes. The model achieved an accuracy of 0.818, with a precision of 0.900, indicating a high proportion of correctly identified positive cases (Table 4). The recall was 0.750, reflecting the model’s ability to detect most true positive cases, and the F1-score was 0.818, confirming a balanced trade-off between precision and recall. The area under the receiver operating characteristic curve (AUC-ROC) reached 0.854 (Figure 15), suggesting strong overall discriminative ability in distinguishing between positive and negative cases.

**Figure 16:**
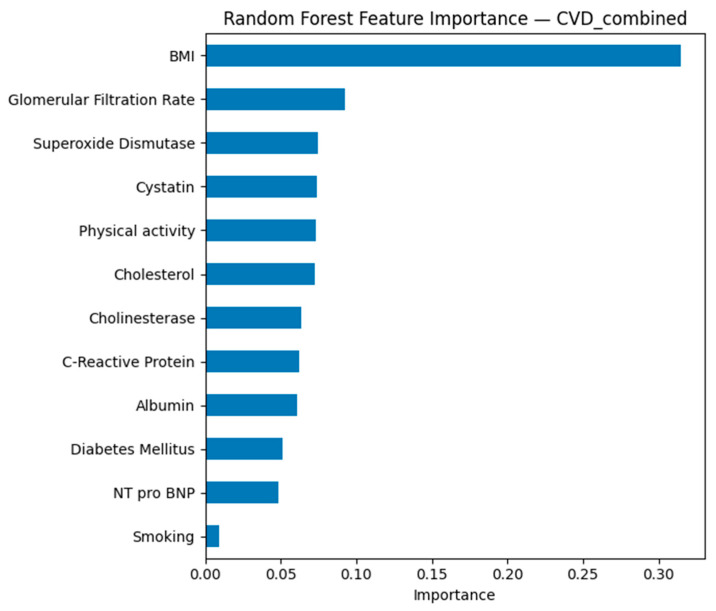
Random Forest feature importance for combined cardiovascular disease (CVD) prediction.

In the integrated model for the combined outcome (CVD overall) in Figure 16, body mass index and glomerular filtration rate were the leading predictors. The high significance of body mass index confirms its universal role in the development of cardiovascular disease. Renal function indicators, including glomerular filtration rate and cystatin, are consistently associated with prognosis. Chemical markers such as cholinesterase, C-reactive protein, and albumin have moderate but reproducible significance, reflecting the role of systemic inflammation and protein metabolism.

The analysis showed that body mass index and arterial hypertension are universal predictors for all outcomes studied. Renal function indicators, including glomerular filtration rate and cystatin, consistently rank among the most significant indicators, especially for stroke and combined cardiovascular outcomes. Cholinesterase demonstrated unexpectedly high significance for stroke, which may indicate the involvement of metabolic and inflammatory mechanisms that require further study. The Random Forest model allowed us to identify priority clinical and laboratory markers, which contributes to improving the effectiveness of screening and prevention of cardiovascular diseases.

The explainable AI analysis using SHAP provided detailed insights into how individual clinical, biochemical, and lifestyle factors contributed to model predictions of cardiovascular aging. The global feature ranking showed that arterial hypertension was by far the most influential predictor (mean |SHAP| = 0.25, 95% CI: 0.24–0.26), followed by body mass index (BMI) (0.07, 95% CI: 0.06–0.08) and physical activity (0.03, 95% CI: 0.02–0.03). These results indicate that elevated blood pressure and increased BMI substantially shift the model’s output toward higher cardiovascular risk, whereas regular physical activity exerts a protective effect.

Among the biochemical indicators, glomerular filtration rate (GFR), cystatin C, and NT-proBNP contributed meaningfully but to a lesser degree (mean |SHAP| ≈ 0.01 each). Their combined influence reflects the role of renal dysfunction and subclinical myocardial stress in premature cardiovascular aging. The inclusion of cholinesterase among relevant predictors suggests a possible link between metabolic regulation and vascular function, warranting further investigation.

**Figure 17:**
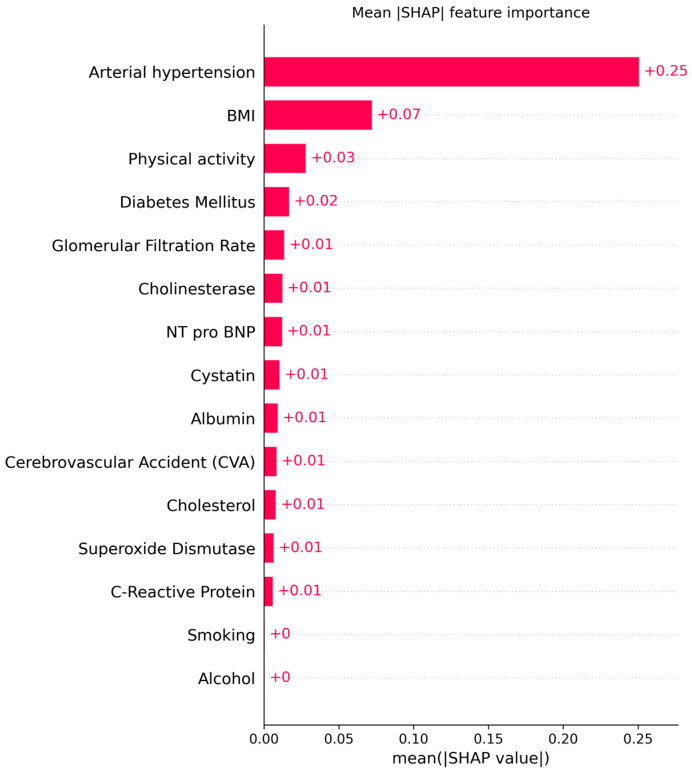
Mean absolute SHAP feature importance with 95% CI.

**Figure 18:**
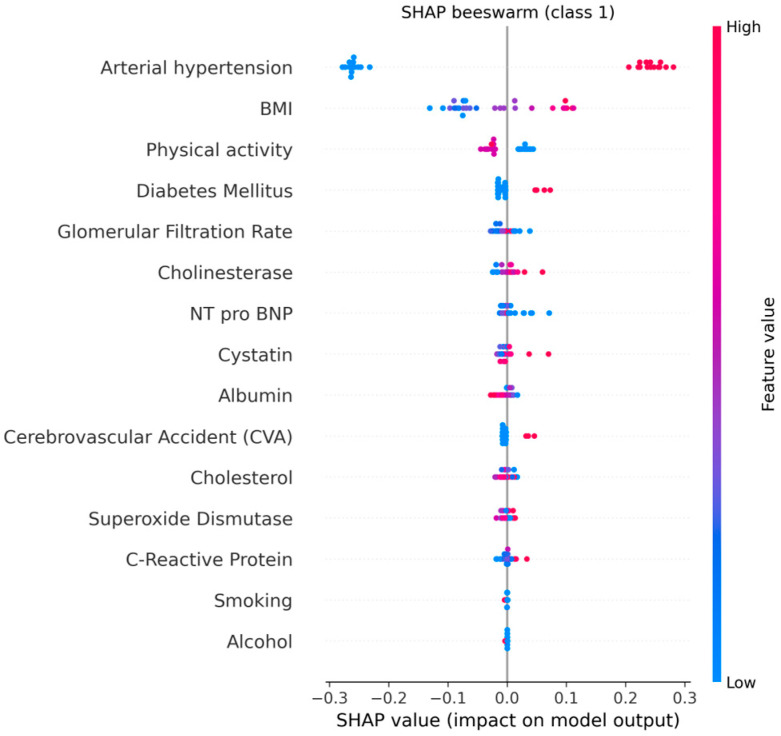
SHAP Beeswarm Plot.

Figure 17 and Figure 18 illustrates the global mean absolute SHAP importance with bootstrap-derived 95% confidence intervals, confirming the relative stability of each feature’s contribution across multiple resampling. The narrow confidence bounds for hypertension and BMI indicate consistent importance estimates, while wider intervals for biochemical markers suggest moderate variability due to the limited cohort size.

These explainability results provide transparent evidence of how the model integrates multiple clinical and biochemical domains to generate individualized risk predictions. By quantifying both the magnitude and direction of each factor’s impact, SHAP interpretation enhances clinical understanding and supports trust in AI-based decision tools.

Overall, these findings confirm the utility of integrating explainable AI methods into cardiovascular research. The SHAP-based interpretation not only identified the most critical determinants of premature cardiovascular aging but also revealed the synergistic interplay between behavioral, clinical, and biochemical domains. The model’s transparency highlights how arterial hypertension, obesity, and sedentary behavior collectively accelerate vascular aging, while preserved renal function and higher physical activity mitigate risk. By combining interpretable machine learning with multidimensional visualization, this framework enables a more comprehensive understanding of biomarker interdependencies and provides an interpretable foundation for future clinical decision support systems.

The integration of biochemical, clinical, and lifestyle features in our AI-based predictive model for combined cardiovascular outcomes (arterial hypertension, ischemic heart disease, and cerebrovascular accidents) yielded robust results, with an AUC-ROC of 0.854 and balanced performance across accuracy (0.818), precision (0.900), and F1-score (0.818). The high precision indicates that the model maintains a low false-positive rate, while the recall (0.750) shows good, though improvable, sensitivity in detecting high-risk individuals.

The explainable AI analysis using SHAP provided transparent feature attribution, revealing that key predictors—BMI, cholesterol, physical activity, superoxide dismutase (SOD), glomerular filtration rate (GFR), cystatin C, and C-reactive protein (CRP)—are consistent with well-established cardiovascular risk factors. These findings align with the existing literature emphasizing the interplay between metabolic health, oxidative stress, renal function, and systemic inflammation in vascular aging. Importantly, SHAP-derived confidence intervals confirmed the stability of feature contributions, with narrow bounds observed for hypertension and BMI, and wider intervals for biochemical indicators, reflecting biological variability.

Our framework’s main strength lies in its multidimensional and interpretable design, combining traditional risk factors with emerging biomarkers to capture the complexity of premature cardiovascular aging. The integration of multimodal visualization (parallel coordinates, PCA, t-SNE) with Random Forest feature ranking enabled simultaneous pattern discovery and predictive interpretation. These complementary methods revealed coherent biomarker-lifestyle profiles across disease states and provided intuitive representations that enhance model explainability.

This study applied multidimensional visualization and AI-based analysis to characterize biomarker, lifestyle, and clinical patterns associated with premature cardiovascular aging, focusing on three outcomes: arterial hypertension, IHD/stent, and CVA.

**Figure 19:**
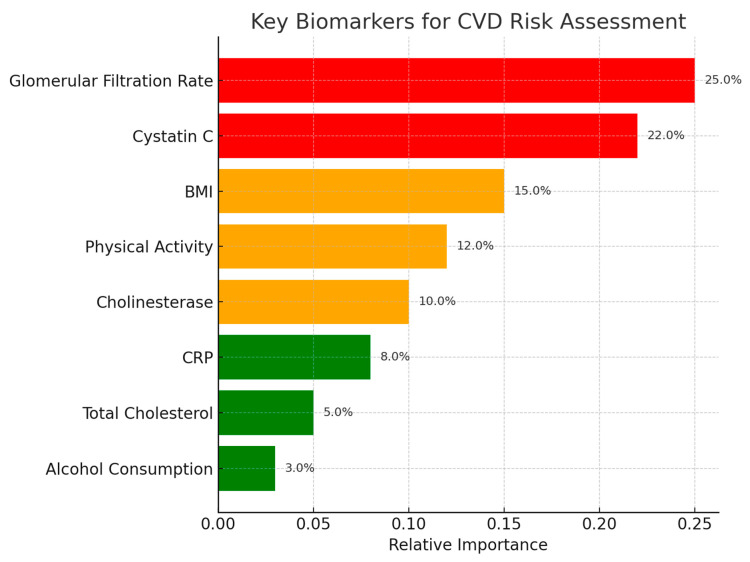
Key patterns for CVD.

Our findings confirm the central role of renal function decline—reflected in reduced GFR and elevated cystatin C—as a shared determinant across outcomes (Figure 19). This aligns with prior evidence linking chronic kidney disease to accelerated vascular aging and increased CVD risk. Physical inactivity emerged as another consistent factor, underscoring the importance of modifiable behavioral components in slowing cardiovascular aging.

Hypertension appeared as a pivotal intermediate stage, nearly universal among patients with IHD/stent and CVA, supporting the “vascular continuum” model. Distinct biomarker patterns, such as higher CRP and cholesterol in IHD/stent and elevated cholinesterase in CVA, suggest differential pathophysiological pathways despite overlapping risk profiles.

The combination of parallel coordinates, t-SNE, PCA, and Random Forest provided both interpretability and predictive insight. Visual methods captured the high-dimensional structure of the data, while AI-based feature ranking identified clinically relevant predictors that could inform targeted prevention.

Nevertheless, several limitations should be noted. The study’s cross-sectional and single-center design limits causal inference, making it impossible to determine whether certain biomarkers act as causes or consequences of cardiovascular aging. The small sample size (*n* = 106) restricts generalizability and statistical power, increasing the potential for overfitting. Confounding variables—such as medication use, comorbidities, or selection bias—may have influenced feature importance estimates.

## 4. Conclusions

This study demonstrates the potential of an integrated, interpretable AI framework for the multidimensional analysis of cardiovascular diseases—specifically arterial hypertension, ischemic heart disease with stent placement, and cerebrovascular accident. By combining Parallel Coordinates, t-distributed Stochastic Neighbor Embedding (t-SNE), and Principal Component Analysis (PCA) with Random Forest–based feature importance and SHAP analysis, the model achieved both high predictive accuracy (AUC-ROC = 0.854, Accuracy = 0.818, Precision = 0.900, Recall = 0.750, F1-score = 0.818) and clinically relevant interpretability.

Visualization of the multidimensional feature space revealed overlapping yet distinct biomarker–lifestyle profiles for each cardiovascular outcome. Parallel Coordinates analysis highlighted consistent trends of reduced glomerular filtration rate, elevated cystatin C levels, and lower physical activity among affected individuals. t-SNE and PCA projections confirmed partial separability across disease types: IHD/stent cases formed the most distinct clusters, hypertension showed moderate differentiation, while CVA cases exhibited greater overlap, reflecting their heterogeneous pathophysiology.

Importantly, t-SNE with k-means clustering identified two sharply distinct participant groups—one exclusively composed of CVD-positive individuals (100%), and another predominantly CVD-negative (7.8%)—demonstrating the unsupervised discriminative power of the selected biomarker set and its potential for early risk identification.

Random Forest and SHAP-based feature attribution revealed arterial hypertension and BMI as the most influential predictors, reaffirming their universal role in cardiovascular aging. Renal function markers—notably GFR and cystatin C—consistently ranked among top contributors, particularly for CVA and combined cardiovascular outcomes. Cholinesterase emerged as an unexpectedly strong determinant of CVA risk, suggesting metabolic and neuroinflammatory involvement. The inclusion of bootstrap confidence intervals for SHAP values enhanced interpretability and quantified model uncertainty, strengthening the robustness of conclusions despite the modest sample size.

By integrating machine learning performance, visual analytics, and explainable AI, this framework bridges the gap between predictive modeling and clinical decision-making. The ability to identify both shared and outcome-specific biomarker patterns provides a foundation for personalized risk stratification, targeted prevention, and early intervention. These findings reinforce the interconnected pathophysiology of cardiovascular and renal systems and highlight the necessity of multidimensional, transparent AI tools for precision cardiovascular medicine.

Despite limitations, this study demonstrates the feasibility and value of combining explainable AI with multidimensional biomarker integration. The framework enhances transparency, supports personalized risk interpretation, and paves the way for precision prevention in cardiovascular aging research. Future work should include multi-center validation, longitudinal follow-up, and integration of imaging and genomic biomarkers to confirm robustness and improve clinical applicability.

## Figures and Tables

**Table 1:** Biochemical markers.

Cholesterol	Total serum cholesterol, a key lipid metabolism component involved in atherosclerotic plaque formation and a major cardiovascular risk factor
Albumin	The main plasma protein reflecting hepatic protein synthesis and nutritional status; decreased levels are associated with inflammation and adverse cardiovascular outcomes.
C-reactive protein (CRP)	An acute-phase inflammatory protein; elevated values indicate systemic inflammation and higher atherothrombotic risk.
Cholinesterase	An enzyme synthesized in the liver that hydrolyzes choline esters; reduced activity may indicate inflammatory or metabolic disorders.
NT-proBNP	N-terminal pro–B-type natriuretic peptide, secreted by cardiomyocytes in response to pressure or volume overload; a biomarker of heart failure.
Cystatin C	A low-molecular-weight inhibitor of cysteine proteases; a sensitive marker of renal function, elevated when glomerular filtration declines.
Glomerular filtration rate (GFR)	An estimate of kidney filtration capacity; reduced GFR is linked to higher cardiovascular risk.
Superoxide dismutase (SOD)	An antioxidant enzyme that catalyzes the dismutation of superoxide radicals; low activity reflects oxidative stress.

**Table 2:** Clinical and social biomarkers.

Smoking	Active tobacco use
Alcohol consumption	Regular alcohol intake
Physical activity	The level of regular exercise
Arterial hypertension	Chronic elevation of blood pressure
Ischemic heart disease/stent placement (IHD/stent)	Coronary artery disease often managed with coronary stenting;
Cerebrovascular accident (CVA)	Acute disruption of cerebral blood flow (stroke)
Diabetes	Chronic hyperglycemia caused by impaired insulin secretion or action

**Table 3:** Distribution of CVD cases across t-SNE–derived clusters.

Cluster	*n*	CVD-Positive	CVD Rate (%)
0	51	4	7.8
1	55	55	100.0

**Table 4:** Evaluation results of the Random Forest model for predicting premature cardiovascular aging.

Accuracy	0.818
Precision	0.9
Recall	0.75
F1-score	0.818
AUC-ROC	0.854

## Data Availability

The presented data in this study are not publicly available due to ongoing research, ethical restrictions, and the need to protect the confidentiality of study participants.
